# Supplementary material for: Individual differences in personality predict the use and perceived effectiveness of essential oils
Source: PLoS One. 2020 Mar 12;15(3):e0229779. doi: 10.1371/journal.pone.0229779 (PMC7067385; doi:10.1371/journal.pone.0229779)
Supplement: S1 Table — (DOCX) [file pone.0229779.s001.docx]

| Supplementary Table 1. Models predicting whether people ever use essential oils | | | | | |
| --- | --- | --- | --- | --- | --- |
|  | *b* | SE | Wald | *p* | Exp(*b*) |
| Intercept | -0.73 | 0.98 | 0.56 | 0.46 | 0.48 |
| Extraversion | 0.28 | 0.13 | 4.66 | 0.03 | 1.32 |
| Agreeableness | -0.17 | 0.14 | 1.47 | 0.23 | 0.85 |
| Conscientiousness | -0.25 | 0.14 | 3.45 | 0.06 | 0.78 |
| Neuroticism | 0.21 | 0.12 | 2.90 | 0.09 | 1.23 |
| Openness to Experience | -0.02 | 0.15 | 0.03 | 0.87 | 0.98 |
| Bullshit Receptivity | 0.24 | 0.10 | 6.15 | 0.01 | 1.27 |
| Need for Cognition | 0.22 | 0.12 | 3.12 | 0.08 | 1.24 |
| Age | -0.01 | 0.01 | 2.16 | 0.14 | 0.99 |
| Gender | -0.50 | 0.08 | 36.16 | <0.001 | 0.61 |
| Income | 0.01 | 0.04 | 0.06 | 0.80 | 1.01 |
| Religiosity | 0.24 | 0.04 | 36.58 | <0.001 | 1.28 |
| Political Orientation | -0.05 | 0.05 | 1.11 | 0.29 | 0.95 |
| Note. Χ2(12) = 149.09. Nagelkerke R2 = .19. | | |  |  |  |
