# Supplementary material for: Individual differences in personality predict the use and perceived effectiveness of essential oils
Source: PLoS One. 2020 Mar 12;15(3):e0229779. doi: 10.1371/journal.pone.0229779 (PMC7067385; doi:10.1371/journal.pone.0229779)
Supplement: S2 Table — (DOCX) [file pone.0229779.s002.docx]

| Supplementary Table 2. Models predicting whether people currently use essential oils | | | | | |
| --- | --- | --- | --- | --- | --- |
|  | *b* | SE | Wald | *p* | Exp(*b*) |
| Intercept | -1.34 | 1.11 | 1.45 | 0.23 | 0.26 |
| Extraversion | 0.22 | 0.15 | 2.20 | 0.14 | 1.24 |
| Agreeableness | -0.10 | 0.16 | 0.39 | 0.53 | 0.90 |
| Conscientiousness | 0.30 | 0.16 | 3.61 | 0.06 | 1.35 |
| Neuroticism | 0.07 | 0.13 | 0.32 | 0.57 | 1.08 |
| Openness to Experience | -0.38 | 0.17 | 5.18 | 0.02 | 0.68 |
| Bullshit Receptivity | 0.53 | 0.11 | 23.63 | <0.001 | 1.70 |
| Need for Cognition | 0.005 | 0.14 | 0.001 | 0.97 | 1.00 |
| Age | 0.01 | 0.01 | 0.88 | 0.35 | 1.01 |
| Gender | 0.15 | 0.10 | 2.21 | 0.14 | 1.16 |
| Income | -0.06 | 0.04 | 2.22 | 0.14 | 0.94 |
| Religiosity | 0.17 | 0.05 | 13.64 | <0.001 | 1.18 |
| Political Orientation | -0.05 | 0.05 | 1.12 | 0.29 | 0.95 |
| Note. Χ2(12) = 96.53. Nagelkerke R2 = .17. | | |  |  |  |
