# Supplementary material for: Individual differences in personality predict the use and perceived effectiveness of essential oils
Source: PLoS One. 2020 Mar 12;15(3):e0229779. doi: 10.1371/journal.pone.0229779 (PMC7067385; doi:10.1371/journal.pone.0229779)
Supplement: S3 Table — (DOCX) [file pone.0229779.s003.docx]

| Supplementary Table 3. Models predicting whether people currently use essential oils topically | | | | | |
| --- | --- | --- | --- | --- | --- |
|  | *b* | SE | Wald | *p* | Exp(*b*) |
| Intercept | -3.27 | 1.07 | 9.32 | 0.002 | 0.04 |
| Extraversion | 0.43 | 0.15 | 8.30 | 0.004 | 1.54 |
| Agreeableness | -0.33 | 0.15 | 5.12 | 0.02 | 0.72 |
| Conscientiousness | 0.10 | 0.14 | 0.50 | 0.48 | 1.11 |
| Neuroticism | 0.27 | 0.13 | 4.11 | 0.04 | 1.30 |
| Openness to Experience | 0.004 | 0.15 | <0.001 | 0.98 | 1.00 |
| Bullshit Receptivity | 0.21 | 0.10 | 4.44 | 0.04 | 1.24 |
| Need for Cognition | -0.05 | 0.13 | 0.15 | 0.70 | 0.95 |
| Age | 0.01 | 0.01 | 1.53 | 0.22 | 1.01 |
| Gender | -0.23 | 0.08 | 7.42 | 0.01 | 0.79 |
| Income | 0.06 | 0.04 | 2.26 | 0.13 | 1.06 |
| Religiosity | 0.15 | 0.04 | 13.87 | <0.001 | 1.16 |
| Political Orientation | -0.09 | 0.04 | 4.46 | 0.03 | 0.91 |
| Note. Χ2(12) = 63.48. Nagelkerke R2 = .10. | | |  |  |  |
