# Supplementary material for: Individual differences in personality predict the use and perceived effectiveness of essential oils
Source: PLoS One. 2020 Mar 12;15(3):e0229779. doi: 10.1371/journal.pone.0229779 (PMC7067385; doi:10.1371/journal.pone.0229779)
Supplement: S4 Table — (DOCX) [file pone.0229779.s004.docx]

| Supplementary Table 4. Models predicting whether people currently use essential oils internally | | | | | |
| --- | --- | --- | --- | --- | --- |
|  | *b* | SE | Wald | *p* | Exp(*b*) |
| Intercept | -0.90 | 1.59 | 0.32 | 0.57 | 0.41 |
| Extraversion | 0.16 | 0.24 | 0.45 | 0.50 | 1.18 |
| Agreeableness | -0.38 | 0.23 | 2.80 | 0.09 | 0.68 |
| Conscientiousness | -0.41 | 0.22 | 3.68 | 0.06 | 0.66 |
| Neuroticism | 0.16 | 0.21 | 0.61 | 0.43 | 1.17 |
| Openness to Experience | -0.85 | 0.23 | 13.27 | <0.001 | 0.43 |
| Bullshit Receptivity | 0.49 | 0.15 | 10.32 | 0.001 | 1.64 |
| Need for Cognition | 0.47 | 0.22 | 4.62 | 0.03 | 1.59 |
| Age | -0.01 | 0.01 | 1.45 | 0.23 | 0.99 |
| Gender | 0.18 | 0.11 | 2.38 | 0.12 | 1.19 |
| Income | 0.01 | 0.05 | 0.01 | 0.91 | 1.01 |
| Religiosity | 0.35 | 0.06 | 31.45 | <0.001 | 1.42 |
| Political Orientation | -0.05 | 0.06 | 0.77 | 0.38 | 0.95 |
| Note. Χ2(12) = 193.81. Nagelkerke R2 = .34. | | |  |  |  |
