# Supplementary material for: Individual differences in personality predict the use and perceived effectiveness of essential oils
Source: PLoS One. 2020 Mar 12;15(3):e0229779. doi: 10.1371/journal.pone.0229779 (PMC7067385; doi:10.1371/journal.pone.0229779)
Supplement: S5 Table — (DOCX) [file pone.0229779.s005.docx]

| Supplementary Table 5. Models predicting whether people currently use essential oils by diffusion | | | | | |
| --- | --- | --- | --- | --- | --- |
|  | *b* | SE | Wald | *p* | Exp(*b*) |
| Intercept | -5.92 | 1.12 | 27.78 | <0.001 | 0.003 |
| Extraversion | 0.38 | 0.15 | 6.39 | 0.01 | 1.46 |
| Agreeableness | 0.31 | 0.15 | 4.15 | 0.04 | 1.36 |
| Conscientiousness | 0.09 | 0.15 | 0.40 | 0.53 | 1.10 |
| Neuroticism | 0.38 | 0.13 | 7.99 | 0.005 | 1.46 |
| Openness to Experience | 0.30 | 0.16 | 3.71 | 0.05 | 1.35 |
| Bullshit Receptivity | -0.13 | 0.10 | 1.62 | 0.20 | 0.88 |
| Need for Cognition | 0.08 | 0.14 | 0.35 | 0.56 | 1.08 |
| Age | -0.02 | 0.01 | 7.86 | 0.01 | 0.98 |
| Gender | -0.39 | 0.09 | 19.38 | <0.001 | 0.68 |
| Income | 0.08 | 0.04 | 4.44 | 0.04 | 1.08 |
| Religiosity | 0.08 | 0.04 | 3.70 | 0.05 | 1.09 |
| Political Orientation | 0.06 | 0.05 | 1.51 | 0.22 | 1.06 |
| Note. Χ2(12) = 112.95. Nagelkerke R2 = .18. | | |  |  |  |
