# Supplementary material for: Individual differences in personality predict the use and perceived effectiveness of essential oils
Source: PLoS One. 2020 Mar 12;15(3):e0229779. doi: 10.1371/journal.pone.0229779 (PMC7067385; doi:10.1371/journal.pone.0229779)
Supplement: S6 Table — (DOCX) [file pone.0229779.s006.docx]

| Supplementary Table 6. Models predicting whether people currently use essential oils for physical ailments | | | | | |
| --- | --- | --- | --- | --- | --- |
|  | *b* | SE | Wald | *p* | Exp(*b*) |
| Intercept | -0.67 | 1.10 | 0.36 | 0.55 | 0.51 |
| Extraversion | 0.08 | 0.15 | 0.31 | 0.58 | 1.08 |
| Agreeableness | -0.06 | 0.16 | 0.15 | 0.70 | 0.94 |
| Conscientiousness | -0.09 | 0.16 | 0.33 | 0.57 | 0.91 |
| Neuroticism | 0.11 | 0.13 | 0.79 | 0.38 | 1.12 |
| Openness to Experience | -0.03 | 0.17 | 0.03 | 0.86 | 0.97 |
| Bullshit Receptivity | 0.30 | 0.11 | 7.63 | 0.01 | 1.35 |
| Need for Cognition | -0.06 | 0.14 | 0.20 | 0.66 | 0.94 |
| Age | 0.01 | 0.01 | 0.66 | 0.42 | 1.01 |
| Gender | 0.20 | 0.10 | 3.95 | 0.05 | 1.22 |
| Income | 0.001 | 0.04 | <0.001 | 0.98 | 1.00 |
| Religiosity | 0.17 | 0.05 | 14.26 | <0.001 | 1.19 |
| Political Orientation | 0.06 | 0.05 | 1.26 | 0.26 | 1.06 |
| Note. Χ2(12) = 63.09. Nagelkerke R2 = .11. | | |  |  |  |
