# Supplementary material for: Individual differences in personality predict the use and perceived effectiveness of essential oils
Source: PLoS One. 2020 Mar 12;15(3):e0229779. doi: 10.1371/journal.pone.0229779 (PMC7067385; doi:10.1371/journal.pone.0229779)
Supplement: S7 Table — (DOCX) [file pone.0229779.s007.docx]

| Supplementary Table 7. Models predicting whether people currently use essential oils to alter mental/emotional state | | | | | |
| --- | --- | --- | --- | --- | --- |
|  | *b* | SE | Wald | *p* | Exp(*b*) |
| Intercept | -1.35 | 1.13 | 1.43 | 0.23 | 0.26 |
| Extraversion | 0.29 | 0.15 | 3.62 | 0.06 | 1.33 |
| Agreeableness | 0.09 | 0.17 | 0.27 | 0.60 | 1.09 |
| Conscientiousness | -0.28 | 0.16 | 2.85 | 0.09 | 0.76 |
| Neuroticism | 0.35 | 0.13 | 6.63 | 0.01 | 1.42 |
| Openness to Experience | 0.05 | 0.17 | 0.08 | 0.77 | 1.05 |
| Bullshit Receptivity | 0.32 | 0.11 | 8.59 | 0.003 | 1.37 |
| Need for Cognition | -0.12 | 0.15 | 0.63 | 0.43 | 0.89 |
| Age | -0.01 | 0.01 | 2.35 | 0.13 | 0.99 |
| Gender | -0.07 | 0.10 | 0.59 | 0.44 | 0.93 |
| Income | 0.05 | 0.04 | 1.64 | 0.20 | 1.05 |
| Religiosity | 0.11 | 0.05 | 5.79 | 0.02 | 1.12 |
| Political Orientation | -0.03 | 0.05 | 0.29 | 0.59 | 0.97 |
| Note. Χ2(12) = 59.30. Nagelkerke R2 = .10. | | |  |  |  |
