# Supplementary material for: Individual differences in personality predict the use and perceived effectiveness of essential oils
Source: PLoS One. 2020 Mar 12;15(3):e0229779. doi: 10.1371/journal.pone.0229779 (PMC7067385; doi:10.1371/journal.pone.0229779)
Supplement: S8 Table — (DOCX) [file pone.0229779.s008.docx]

| Supplementary Table 8. Models predicting whether people currently use essential oils for avoiding physical/mental illness | | | | | |
| --- | --- | --- | --- | --- | --- |
|  | *b* | SE | Wald | *p* | Exp(*b*) |
| Intercept | 0.80 | 1.28 | 0.39 | 0.53 | 2.23 |
| Extraversion | 0.29 | 0.18 | 2.57 | 0.11 | 1.33 |
| Agreeableness | -0.43 | 0.19 | 5.16 | 0.02 | 0.65 |
| Conscientiousness | -0.48 | 0.18 | 6.84 | 0.01 | 0.62 |
| Neuroticism | -0.14 | 0.16 | 0.83 | 0.36 | 0.87 |
| Openness to Experience | -0.62 | 0.19 | 10.92 | <0.001 | 0.54 |
| Bullshit Receptivity | 0.67 | 0.13 | 27.27 | <0.001 | 1.96 |
| Need for Cognition | 0.14 | 0.17 | 0.70 | 0.40 | 1.15 |
| Age | 0.00 | 0.01 | 0.07 | 0.79 | 1.00 |
| Gender | 0.20 | 0.10 | 3.73 | 0.05 | 1.22 |
| Income | 0.00 | 0.04 | 0.002 | 0.96 | 1.00 |
| Religiosity | 0.29 | 0.05 | 30.77 | <0.001 | 1.33 |
| Political Orientation | -0.07 | 0.05 | 1.55 | 0.21 | 0.94 |
| Note. Χ2(12) = 256.27. Nagelkerke R2 = .39. | | |  |  |  |
