# Supplementary material for: Individual differences in personality predict the use and perceived effectiveness of essential oils
Source: PLoS One. 2020 Mar 12;15(3):e0229779. doi: 10.1371/journal.pone.0229779 (PMC7067385; doi:10.1371/journal.pone.0229779)
Supplement: S9 Table — (DOCX) [file pone.0229779.s009.docx]

| Supplementary Table 9. Models predicting whether people currently use essential oils to improve mood | | | | | |
| --- | --- | --- | --- | --- | --- |
|  | *b* | SE | Wald | *p* | Exp(*b*) |
| Intercept | -1.19 | 1.09 | 1.20 | 0.27 | 0.30 |
| Extraversion | 0.07 | 0.14 | 0.24 | 0.63 | 1.07 |
| Agreeableness | -0.001 | 0.16 | 0.00 | 0.99 | 1.00 |
| Conscientiousness | -0.23 | 0.16 | 2.08 | 0.15 | 0.80 |
| Neuroticism | 0.19 | 0.13 | 2.25 | 0.13 | 1.21 |
| Openness to Experience | 0.12 | 0.16 | 0.56 | 0.45 | 1.13 |
| Bullshit Receptivity | 0.48 | 0.11 | 19.98 | <0.001 | 1.61 |
| Need for Cognition | -0.09 | 0.14 | 0.46 | 0.50 | 0.91 |
| Age | -0.001 | 0.01 | 0.05 | 0.83 | 1.00 |
| Gender | 0.01 | 0.09 | 0.01 | 0.91 | 1.01 |
| Income | -0.004 | 0.04 | 0.01 | 0.91 | 1.00 |
| Religiosity | 0.08 | 0.04 | 3.26 | 0.07 | 1.08 |
| Political Orientation | 0.07 | 0.05 | 1.94 | 0.16 | 1.07 |
| Note. Χ2(12) = 57.46. Nagelkerke R2 = .10. | | |  |  |  |
