# Supplementary material for: Individual differences in personality predict the use and perceived effectiveness of essential oils
Source: PLoS One. 2020 Mar 12;15(3):e0229779. doi: 10.1371/journal.pone.0229779 (PMC7067385; doi:10.1371/journal.pone.0229779)
Supplement: S10 Table — (DOCX) [file pone.0229779.s010.docx]

| Supplementary Table 10. Models predicting whether people currently use essential oils to help sleep | | | | | |
| --- | --- | --- | --- | --- | --- |
|  | *b* | SE | Wald | *p* | Exp(*b*) |
| Intercept | -0.10 | 1.06 | 0.01 | 0.93 | 0.91 |
| Extraversion | 0.28 | 0.14 | 3.72 | 0.05 | 1.32 |
| Agreeableness | 0.23 | 0.16 | 2.10 | 0.15 | 1.26 |
| Conscientiousness | -0.45 | 0.15 | 8.46 | 0.004 | 0.64 |
| Neuroticism | 0.08 | 0.13 | 0.38 | 0.54 | 1.08 |
| Openness to Experience | -0.36 | 0.16 | 5.14 | 0.02 | 0.70 |
| Bullshit Receptivity | 0.39 | 0.10 | 13.97 | <0.001 | 1.47 |
| Need for Cognition | 0.15 | 0.14 | 1.15 | 0.28 | 1.16 |
| Age | -0.01 | 0.01 | 4.94 | 0.03 | 0.99 |
| Gender | -0.06 | 0.09 | 0.46 | 0.50 | 0.94 |
| Income | -0.03 | 0.04 | 0.76 | 0.38 | 0.97 |
| Religiosity | 0.07 | 0.04 | 2.88 | 0.09 | 1.07 |
| Political Orientation | 0.04 | 0.05 | 0.58 | 0.44 | 1.04 |
| Note. Χ2(12) = 80.71. Nagelkerke R2 = .14. | | |  |  |  |
