# Supplementary material for: Individual differences in personality predict the use and perceived effectiveness of essential oils
Source: PLoS One. 2020 Mar 12;15(3):e0229779. doi: 10.1371/journal.pone.0229779 (PMC7067385; doi:10.1371/journal.pone.0229779)
Supplement: S11 Table — (DOCX) [file pone.0229779.s011.docx]

| Supplementary Table 11. Models predicting whether people currently use essential oils to enhance spiritual life | | | | | |
| --- | --- | --- | --- | --- | --- |
|  | *b* | SE | Wald | *p* | Exp(*b*) |
| Intercept | 3.25 | 1.43 | 5.14 | 0.02 | 25.71 |
| Extraversion | -0.14 | 0.20 | 0.49 | 0.49 | 0.87 |
| Agreeableness | -0.74 | 0.21 | 12.25 | <0.001 | 0.48 |
| Conscientiousness | -0.63 | 0.20 | 9.87 | 0.002 | 0.53 |
| Neuroticism | -0.52 | 0.18 | 8.03 | 0.005 | 0.60 |
| Openness to Experience | -0.08 | 0.21 | 0.12 | 0.72 | 0.93 |
| Bullshit Receptivity | 1.06 | 0.15 | 48.93 | <0.001 | 2.88 |
| Need for Cognition | -0.18 | 0.18 | 0.91 | 0.34 | 0.84 |
| Age | 0.01 | 0.01 | 0.93 | 0.33 | 1.01 |
| Gender | 0.02 | 0.11 | 0.05 | 0.83 | 1.02 |
| Income | -0.13 | 0.05 | 8.06 | 0.005 | 0.87 |
| Religiosity | 0.29 | 0.06 | 26.96 | <0.001 | 1.34 |
| Political Orientation | -0.12 | 0.06 | 4.61 | 0.03 | 0.88 |
| Note. Χ2(12) = 311.63. Nagelkerke R2 = .47. | | |  |  |  |
