# Supplementary material for: Individual differences in personality predict the use and perceived effectiveness of essential oils
Source: PLoS One. 2020 Mar 12;15(3):e0229779. doi: 10.1371/journal.pone.0229779 (PMC7067385; doi:10.1371/journal.pone.0229779)
Supplement: S12 Table — (DOCX) [file pone.0229779.s012.docx]

| Supplementary Table 12. Models predicting whether people currently use essential oils to improve/sustain relationships | | | | | |
| --- | --- | --- | --- | --- | --- |
|  | *b* | SE | Wald | *p* | Exp(*b*) |
| Intercept | 2.64 | 1.89 | 1.95 | 0.16 | 14.00 |
| Extraversion | -0.05 | 0.28 | 0.03 | 0.87 | 0.96 |
| Agreeableness | -0.81 | 0.28 | 8.24 | 0.004 | 0.45 |
| Conscientiousness | -0.91 | 0.27 | 11.65 | <0.001 | 0.40 |
| Neuroticism | -0.65 | 0.25 | 6.72 | 0.01 | 0.52 |
| Openness to Experience | -0.89 | 0.28 | 10.14 | 0.001 | 0.41 |
| Bullshit Receptivity | 1.63 | 0.21 | 58.41 | <0.001 | 5.10 |
| Need for Cognition | 0.17 | 0.25 | 0.48 | 0.49 | 1.19 |
| Age | 0.01 | 0.01 | 0.57 | 0.45 | 1.01 |
| Gender | 0.09 | 0.13 | 0.42 | 0.52 | 1.09 |
| Income | -0.07 | 0.06 | 1.24 | 0.27 | 0.94 |
| Religiosity | 0.33 | 0.07 | 21.36 | <0.001 | 1.39 |
| Political Orientation | -0.02 | 0.07 | 0.11 | 0.74 | 0.98 |
| Note. Χ2(12) = 405.91. Nagelkerke R2 = .61. | | |  |  |  |
