# Supplementary material for: Individual differences in personality predict the use and perceived effectiveness of essential oils
Source: PLoS One. 2020 Mar 12;15(3):e0229779. doi: 10.1371/journal.pone.0229779 (PMC7067385; doi:10.1371/journal.pone.0229779)
Supplement: S13 Table — (DOCX) [file pone.0229779.s013.docx]

| Supplementary Table 13. Models predicting whether people currently use essential oils as dietary supplement | | | | | |
| --- | --- | --- | --- | --- | --- |
|  | *b* | SE | Wald | *p* | Exp(*b*) |
| Intercept | 2.33 | 1.72 | 1.82 | 0.18 | 10.25 |
| Extraversion | 0.08 | 0.25 | 0.10 | 0.76 | 1.08 |
| Agreeableness | -0.74 | 0.26 | 8.27 | 0.004 | 0.48 |
| Conscientiousness | -0.75 | 0.25 | 9.26 | 0.002 | 0.47 |
| Neuroticism | -0.46 | 0.23 | 4.06 | 0.04 | 0.63 |
| Openness to Experience | -1.03 | 0.26 | 15.75 | <0.001 | 0.36 |
| Bullshit Receptivity | 1.08 | 0.18 | 37.15 | <0.001 | 2.94 |
| Need for Cognition | 0.49 | 0.24 | 4.37 | 0.04 | 1.64 |
| Age | 0.01 | 0.01 | 0.83 | 0.36 | 1.01 |
| Gender | 0.25 | 0.12 | 4.09 | 0.04 | 1.28 |
| Income | -0.05 | 0.06 | 0.75 | 0.39 | 0.95 |
| Religiosity | 0.29 | 0.07 | 19.90 | <0.001 | 1.34 |
| Political Orientation | -0.03 | 0.06 | 0.25 | 0.62 | 0.97 |
| Note. Χ2(12) = 341.42. Nagelkerke R2 = .53. | | |  |  |  |
