# Supplementary material for: Individual differences in personality predict the use and perceived effectiveness of essential oils
Source: PLoS One. 2020 Mar 12;15(3):e0229779. doi: 10.1371/journal.pone.0229779 (PMC7067385; doi:10.1371/journal.pone.0229779)
Supplement: S14 Table — (DOCX) [file pone.0229779.s014.docx]

| Supplementary Table 14. Models predicting whether people currently use essential oils to clean/disinfect | | | | | |
| --- | --- | --- | --- | --- | --- |
|  | *b* | SE | Wald | *p* | Exp(*b*) |
| Intercept | -0.22 | 1.12 | 0.04 | 0.85 | 0.81 |
| Extraversion | -0.15 | 0.15 | 0.94 | 0.33 | 0.86 |
| Agreeableness | -0.33 | 0.16 | 4.00 | 0.05 | 0.72 |
| Conscientiousness | -0.27 | 0.16 | 2.84 | 0.09 | 0.77 |
| Neuroticism | -0.06 | 0.13 | 0.18 | 0.67 | 0.94 |
| Openness to Experience | -0.26 | 0.17 | 2.45 | 0.12 | 0.77 |
| Bullshit Receptivity | 0.56 | 0.11 | 25.20 | <0.001 | 1.74 |
| Need for Cognition | 0.28 | 0.15 | 3.64 | 0.06 | 1.32 |
| Age | 0.03 | 0.01 | 14.36 | <0.001 | 1.03 |
| Gender | 0.001 | 0.09 | <0.001 | 0.99 | 1.00 |
| Income | -0.04 | 0.04 | 1.38 | 0.24 | 0.96 |
| Religiosity | 0.14 | 0.04 | 9.89 | <0.001 | 1.15 |
| Political Orientation | -0.05 | 0.05 | 0.98 | 0.32 | 0.95 |
| Note. Χ2(12) = 126.29. Nagelkerke R2 = .21. | | |  |  |  |
