# Supplementary material for: Individual differences in personality predict the use and perceived effectiveness of essential oils
Source: PLoS One. 2020 Mar 12;15(3):e0229779. doi: 10.1371/journal.pone.0229779 (PMC7067385; doi:10.1371/journal.pone.0229779)
Supplement: S15 Table — (DOCX) [file pone.0229779.s015.docx]

| Supplementary Table 15. Models predicting the effectiveness of EO for relieving physical ailments | | | | | | | |
| --- | --- | --- | --- | --- | --- | --- | --- |
|  | *b* | SE | *β* | *t* | *p* | LB | UB |
| Intercept | 2.88 | 0.66 |  | 4.34 | <0.001 | 1.58 | 4.19 |
| Extraversion | 0.07 | 0.09 | 0.04 | 0.74 | 0.46 | -0.11 | 0.24 |
| Agreeableness | 0.14 | 0.10 | 0.08 | 1.41 | 0.16 | -0.05 | 0.33 |
| Conscientiousness | -0.10 | 0.09 | -0.06 | -1.13 | 0.26 | -0.29 | 0.08 |
| Neuroticism | 0.03 | 0.08 | 0.02 | 0.38 | 0.70 | -0.12 | 0.18 |
| Openness to Experience | -0.25 | 0.10 | -0.17 | -2.63 | 0.01 | -0.44 | -0.06 |
| Bullshit Receptivity | 0.29 | 0.06 | 0.21 | 4.56 | <0.001 | 0.16 | 0.41 |
| Need for Cognition | 0.03 | 0.08 | 0.02 | 0.39 | 0.70 | -0.13 | 0.19 |
| Age | -0.004 | 0.00 | -0.04 | -1.00 | 0.32 | -0.01 | 0.004 |
| Gender | 0.06 | 0.05 | 0.05 | 1.17 | 0.24 | -0.04 | 0.17 |
| Income | -0.02 | 0.02 | -0.04 | -0.88 | 0.38 | -0.06 | 0.02 |
| Religiosity | 0.08 | 0.03 | 0.14 | 2.92 | 0.004 | 0.02 | 0.13 |
| Political Orientation | -0.05 | 0.03 | -0.09 | -2.09 | 0.04 | -0.10 | -0.003 |
| Note. F(12, 529) = 8.35, p < .001; R2 = .16 | | |  |  |  |  |  |
