# Supplementary material for: Individual differences in personality predict the use and perceived effectiveness of essential oils
Source: PLoS One. 2020 Mar 12;15(3):e0229779. doi: 10.1371/journal.pone.0229779 (PMC7067385; doi:10.1371/journal.pone.0229779)
Supplement: S16 Table — (DOCX) [file pone.0229779.s016.docx]

| Supplementary Table 16. Models predicting the effectiveness of EO for altering mental/emotional state | | | | | | | |
| --- | --- | --- | --- | --- | --- | --- | --- |
|  | *b* | SE | *β* | *t* | *p* | LB | UB |
| Intercept | 3.13 | 0.65 |  | 4.78 | <0.001 | 1.84 | 4.42 |
| Extraversion | 0.12 | 0.09 | 0.07 | 1.32 | 0.19 | -0.06 | 0.31 |
| Agreeableness | -0.06 | 0.10 | -0.03 | -0.59 | 0.55 | -0.24 | 0.13 |
| Conscientiousness | -0.06 | 0.09 | -0.04 | -0.71 | 0.48 | -0.24 | 0.11 |
| Neuroticism | -0.09 | 0.08 | -0.06 | -1.20 | 0.23 | -0.25 | 0.06 |
| Openness to Experience | -0.07 | 0.09 | -0.05 | -0.79 | 0.43 | -0.26 | 0.11 |
| Bullshit Receptivity | 0.24 | 0.06 | 0.18 | 3.67 | <0.001 | 0.11 | 0.36 |
| Need for Cognition | 0.04 | 0.08 | 0.03 | 0.46 | 0.65 | -0.12 | 0.20 |
| Age | -0.01 | 0.004 | -0.09 | -1.90 | 0.06 | -0.02 | <0.001 |
| Gender | 0.03 | 0.05 | 0.02 | 0.51 | 0.61 | -0.08 | 0.13 |
| Income | -0.03 | 0.02 | -0.06 | -1.38 | 0.17 | -0.07 | 0.01 |
| Religiosity | 0.07 | 0.03 | 0.14 | 2.82 | 0.005 | 0.02 | 0.13 |
| Political Orientation | -0.02 | 0.03 | -0.03 | -0.60 | 0.55 | -0.07 | 0.04 |
| Note. F(12, 534) = 6.00, p < .001; R2 = .12 | | |  |  |  |  |  |
