# Supplementary material for: Individual differences in personality predict the use and perceived effectiveness of essential oils
Source: PLoS One. 2020 Mar 12;15(3):e0229779. doi: 10.1371/journal.pone.0229779 (PMC7067385; doi:10.1371/journal.pone.0229779)
Supplement: S17 Table — (DOCX) [file pone.0229779.s017.docx]

| Supplementary Table 17. Models predicting the effectiveness of EO for avoiding physical/mental illness | | | | | | | |
| --- | --- | --- | --- | --- | --- | --- | --- |
|  | *b* | SE | *β* | *t* | *p* | LB | UB |
| Intercept | 2.35 | 1.04 |  | 2.27 | 0.02 | 0.31 | 4.39 |
| Extraversion | 0.07 | 0.16 | 0.03 | 0.40 | 0.69 | -0.26 | 0.39 |
| Agreeableness | -0.02 | 0.18 | -0.01 | -0.13 | 0.89 | -0.37 | 0.32 |
| Conscientiousness | 0.10 | 0.17 | 0.06 | 0.62 | 0.54 | -0.23 | 0.44 |
| Neuroticism | -0.08 | 0.13 | -0.04 | -0.59 | 0.56 | -0.34 | 0.18 |
| Openness to Experience | -0.25 | 0.16 | -0.15 | -1.56 | 0.12 | -0.57 | 0.07 |
| Bullshit Receptivity | 0.27 | 0.10 | 0.19 | 2.66 | 0.01 | 0.07 | 0.47 |
| Need for Cognition | 0.19 | 0.14 | 0.11 | 1.33 | 0.18 | -0.09 | 0.46 |
| Age | 0.004 | 0.01 | 0.03 | 0.57 | 0.57 | -0.01 | 0.02 |
| Gender | -0.01 | 0.07 | -0.01 | -0.12 | 0.90 | -0.14 | 0.13 |
| Income | 0.01 | 0.03 | 0.02 | 0.26 | 0.79 | -0.06 | 0.07 |
| Religiosity | 0.03 | 0.04 | 0.06 | 0.85 | 0.39 | -0.04 | 0.11 |
| Political Orientation | -0.07 | 0.03 | -0.13 | -2.02 | 0.04 | -0.13 | 0.00 |
| Note. F(12, 268) = 1.64, p = .08; R2 = .07 | | |  |  |  |  |  |
