# Supplementary material for: Individual differences in personality predict the use and perceived effectiveness of essential oils
Source: PLoS One. 2020 Mar 12;15(3):e0229779. doi: 10.1371/journal.pone.0229779 (PMC7067385; doi:10.1371/journal.pone.0229779)
Supplement: S18 Table — (DOCX) [file pone.0229779.s018.docx]

| Supplementary Table 18. Models predicting the effectiveness of EO to improve mood | | | | | | |  |
| --- | --- | --- | --- | --- | --- | --- | --- |
|  | *b* | SE | *β* | *t* | *p* | LB | UB |
| Intercept | 4.14 | 0.67 |  | 6.21 | <0.001 | 2.83 | 5.45 |
| Extraversion | 0.07 | 0.09 | 0.04 | 0.82 | 0.42 | -0.11 | 0.25 |
| Agreeableness | -0.04 | 0.10 | -0.03 | -0.43 | 0.66 | -0.23 | 0.15 |
| Conscientiousness | -0.15 | 0.09 | -0.10 | -1.63 | 0.10 | -0.33 | 0.03 |
| Neuroticism | -0.26 | 0.08 | -0.18 | -3.26 | 0.001 | -0.42 | -0.10 |
| Openness to Experience | -0.09 | 0.09 | -0.06 | -0.96 | 0.34 | -0.28 | 0.10 |
| Bullshit Receptivity | 0.23 | 0.06 | 0.18 | 3.69 | <0.001 | 0.11 | 0.36 |
| Need for Cognition | 0.02 | 0.08 | 0.02 | 0.27 | 0.79 | -0.14 | 0.18 |
| Age | -0.01 | 0.004 | -0.08 | -1.62 | 0.11 | -0.01 | 0.001 |
| Gender | -0.01 | 0.05 | -0.01 | -0.24 | 0.81 | -0.12 | 0.09 |
| Income | -0.01 | 0.02 | -0.01 | -0.34 | 0.73 | -0.05 | 0.03 |
| Religiosity | 0.03 | 0.03 | 0.06 | 1.30 | 0.196 | -0.02 | 0.08 |
| Political Orientation | -0.02 | 0.03 | -0.04 | -0.93 | 0.35 | -0.07 | 0.03 |
| Note. F(12, 507) = 5.17, p < .001; R2 = .11 | | |  |  |  |  |  |
