# Supplementary material for: Individual differences in personality predict the use and perceived effectiveness of essential oils
Source: PLoS One. 2020 Mar 12;15(3):e0229779. doi: 10.1371/journal.pone.0229779 (PMC7067385; doi:10.1371/journal.pone.0229779)
Supplement: S19 Table — (DOCX) [file pone.0229779.s019.docx]

| Supplementary Table 19. Models predicting the effectiveness of EO to help sleep | | | | | | |  |
| --- | --- | --- | --- | --- | --- | --- | --- |
|  | *b* | SE | *β* | *t* | *p* | LB | UB |
| Intercept | 2.10 | 0.80 |  | 2.63 | 0.01 | 0.53 | 3.67 |
| Extraversion | 0.28 | 0.11 | 0.14 | 2.44 | 0.01 | 0.05 | 0.50 |
| Agreeableness | 0.04 | 0.12 | 0.02 | 0.32 | 0.75 | -0.19 | 0.27 |
| Conscientiousness | -0.04 | 0.11 | -0.03 | -0.40 | 0.69 | -0.26 | 0.17 |
| Neuroticism | -0.005 | 0.10 | -0.003 | -0.05 | 0.96 | -0.20 | 0.19 |
| Openness to Experience | 0.02 | 0.11 | 0.01 | 0.22 | 0.83 | -0.20 | 0.25 |
| Bullshit Receptivity | 0.18 | 0.07 | 0.13 | 2.49 | 0.01 | 0.04 | 0.33 |
| Need for Cognition | -0.13 | 0.10 | -0.08 | -1.27 | 0.20 | -0.32 | 0.07 |
| Age | -0.01 | 0.005 | -0.12 | -2.50 | 0.01 | -0.02 | 0.00 |
| Gender | 0.08 | 0.06 | 0.07 | 1.34 | 0.18 | -0.04 | 0.20 |
| Income | 0.01 | 0.03 | 0.01 | 0.29 | 0.77 | -0.04 | 0.06 |
| Religiosity | 0.12 | 0.03 | 0.21 | 3.85 | <0.001 | 0.06 | 0.18 |
| Political Orientation | -0.03 | 0.03 | -0.06 | -1.18 | 0.24 | -0.09 | 0.02 |
| Note. F(12, 432) = 5.69, p < .001; R2 = .14 | | |  |  |  |  |  |
