# Supplementary material for: Individual differences in personality predict the use and perceived effectiveness of essential oils
Source: PLoS One. 2020 Mar 12;15(3):e0229779. doi: 10.1371/journal.pone.0229779 (PMC7067385; doi:10.1371/journal.pone.0229779)
Supplement: S20 Table — (DOCX) [file pone.0229779.s020.docx]

| Supplementary Table 20. Models predicting the effectiveness of EO to enhance spiritual life | | | | | | | |
| --- | --- | --- | --- | --- | --- | --- | --- |
|  | *b* | SE | *β* | *t* | *p* | LB | UB |
| Intercept | 3.36 | 1.23 |  | 2.74 | 0.01 | 0.95 | 5.78 |
| Extraversion | -0.45 | 0.20 | -0.19 | -2.29 | 0.02 | -0.83 | -0.06 |
| Agreeableness | -0.27 | 0.19 | -0.14 | -1.41 | 0.16 | -0.65 | 0.11 |
| Conscientiousness | 0.08 | 0.18 | 0.05 | 0.47 | 0.64 | -0.27 | 0.44 |
| Neuroticism | -0.147 | 0.15 | -0.079 | -0.97 | 0.33 | -0.44 | 0.15 |
| Openness to Experience | 0.13 | 0.17 | 0.07 | 0.75 | 0.46 | -0.21 | 0.46 |
| Bullshit Receptivity | 0.21 | 0.11 | 0.14 | 1.87 | 0.06 | -0.01 | 0.44 |
| Need for Cognition | 0.34 | 0.15 | 0.17 | 2.26 | 0.03 | 0.04 | 0.63 |
| Age | 0.01 | 0.006 | 0.07 | 0.97 | 0.33 | -0.01 | 0.02 |
| Gender | -0.10 | 0.07 | -0.09 | -1.37 | 0.17 | -0.24 | 0.04 |
| Income | 0.01 | 0.03 | 0.02 | 0.33 | 0.74 | -0.06 | 0.08 |
| Religiosity | 0.09 | 0.04 | 0.15 | 2.11 | 0.04 | 0.01 | 0.17 |
| Political Orientation | -0.08 | 0.03 | -0.15 | -2.24 | 0.03 | -0.14 | -0.01 |
| Note. F(12, 228) = 2.14, p = .02; R2 = .10 | | |  |  |  |  |  |
