# Supplementary material for: Individual differences in personality predict the use and perceived effectiveness of essential oils
Source: PLoS One. 2020 Mar 12;15(3):e0229779. doi: 10.1371/journal.pone.0229779 (PMC7067385; doi:10.1371/journal.pone.0229779)
Supplement: S21 Table — (DOCX) [file pone.0229779.s021.docx]

| Supplementary Table 21. Models predicting the effectiveness of EO to sustain/improve relationships | | | | | | | |
| --- | --- | --- | --- | --- | --- | --- | --- |
|  | *b* | SE | *β* | *t* | *p* | LB | UB |
| Intercept | 3.52 | 1.77 |  | 1.99 | 0.05 | 0.03 | 7.00 |
| Extraversion | 0.28 | 0.30 | 0.08 | 0.93 | 0.35 | -0.32 | 0.88 |
| Agreeableness | -0.60 | 0.27 | -0.26 | -2.21 | 0.03 | -1.13 | -0.06 |
| Conscientiousness | -0.09 | 0.27 | -0.04 | -0.32 | 0.75 | -0.62 | 0.45 |
| Neuroticism | -0.042 | 0.27 | -0.014 | -0.15 | 0.88 | -0.58 | 0.50 |
| Openness to Experience | 0.37 | 0.26 | 0.14 | 1.45 | 0.15 | -0.13 | 0.88 |
| Bullshit Receptivity | 0.10 | 0.15 | 0.05 | 0.69 | 0.49 | -0.19 | 0.39 |
| Need for Cognition | -0.12 | 0.22 | -0.05 | -0.57 | 0.57 | -0.55 | 0.30 |
| Age | -0.01 | 0.008 | -0.05 | -0.72 | 0.47 | -0.02 | 0.01 |
| Gender | -0.09 | 0.08 | -0.08 | -1.11 | 0.27 | -0.24 | 0.07 |
| Income | 0.004 | 0.04 | 0.01 | 0.09 | 0.93 | -0.08 | 0.08 |
| Religiosity | 0.15 | 0.05 | 0.22 | 2.71 | 0.007 | 0.04 | 0.25 |
| Political Orientation | -0.12 | 0.04 | -0.24 | -3.22 | 0.002 | -0.19 | -0.05 |
| Note. F(12, 176) = 2.50, p = .005; R2 = .15 | | |  |  |  |  |  |
