# Supplementary material for: Individual differences in personality predict the use and perceived effectiveness of essential oils
Source: PLoS One. 2020 Mar 12;15(3):e0229779. doi: 10.1371/journal.pone.0229779 (PMC7067385; doi:10.1371/journal.pone.0229779)
Supplement: S22 Table — (DOCX) [file pone.0229779.s022.docx]

| Supplementary Table 22. Models predicting the effectiveness of EO to clean/disinfect | | | | | | |  |
| --- | --- | --- | --- | --- | --- | --- | --- |
|  | *b* | SE | *β* | *t* | *p* | LB | UB |
| Intercept | 2.00 | 0.94 |  | 2.13 | 0.03 | 0.16 | 3.85 |
| Extraversion | -0.05 | 0.13 | -0.03 | -0.37 | 0.71 | -0.30 | 0.20 |
| Agreeableness | 0.09 | 0.14 | 0.05 | 0.61 | 0.54 | -0.19 | 0.36 |
| Conscientiousness | 0.02 | 0.14 | 0.02 | 0.17 | 0.86 | -0.24 | 0.29 |
| Neuroticism | -0.02 | 0.12 | -0.01 | -0.14 | 0.89 | -0.25 | 0.22 |
| Openness to Experience | 0.03 | 0.13 | 0.02 | 0.24 | 0.81 | -0.22 | 0.29 |
| Bullshit Receptivity | 0.14 | 0.08 | 0.12 | 1.76 | 0.08 | -0.02 | 0.31 |
| Need for Cognition | 0.16 | 0.12 | 0.11 | 1.38 | 0.17 | -0.07 | 0.39 |
| Age | 0.01 | 0.01 | 0.08 | 1.33 | 0.18 | 0.00 | 0.02 |
| Gender | -0.11 | 0.07 | -0.10 | -1.63 | 0.10 | -0.23 | 0.02 |
| Income | 0.01 | 0.03 | 0.02 | 0.33 | 0.74 | -0.05 | 0.07 |
| Religiosity | -0.003 | 0.03 | -0.01 | -0.09 | 0.93 | -0.07 | 0.06 |
| Political Orientation | 0.03 | 0.03 | 0.06 | 1.08 | 0.28 | -0.03 | 0.09 |
| Note. F(12, 306) = 1.37, p = .178; R2 = .05 | | |  |  |  |  |  |
