# Supplementary material for: Individual differences in personality predict the use and perceived effectiveness of essential oils
Source: PLoS One. 2020 Mar 12;15(3):e0229779. doi: 10.1371/journal.pone.0229779 (PMC7067385; doi:10.1371/journal.pone.0229779)
Supplement: S23 Table — (DOCX) [file pone.0229779.s023.docx]

| Supplementary Table 23. Models predicting the effectiveness of EO as a dietary supplement | | | | | | | |
| --- | --- | --- | --- | --- | --- | --- | --- |
|  | *b* | SE | *β* | *t* | *p* | LB | UB |
| Intercept | 2.10 | 1.56 |  | 1.34 | 0.181 | -0.98 | 5.17 |
| Extraversion | -0.22 | 0.23 | -0.07 | -0.93 | 0.35 | -0.68 | 0.24 |
| Agreeableness | -0.27 | 0.22 | -0.12 | -1.24 | 0.22 | -0.71 | 0.16 |
| Conscientiousness | 0.03 | 0.21 | 0.02 | 0.17 | 0.87 | -0.37 | 0.44 |
| Neuroticism | 0.41 | 0.21 | 0.16 | 1.99 | 0.05 | 0.00 | 0.81 |
| Openness to Experience | 0.07 | 0.21 | 0.03 | 0.31 | 0.76 | -0.36 | 0.49 |
| Bullshit Receptivity | 0.48 | 0.13 | 0.30 | 3.82 | <0.001 | 0.23 | 0.73 |
| Need for Cognition | 0.04 | 0.20 | 0.02 | 0.22 | 0.83 | -0.35 | 0.44 |
| Age | -0.02 | 0.01 | -0.19 | -2.74 | 0.01 | -0.03 | -0.01 |
| Gender | 0.01 | 0.08 | 0.01 | 0.11 | 0.92 | -0.14 | 0.16 |
| Income | 0.05 | 0.04 | 0.10 | 1.40 | 0.16 | -0.02 | 0.12 |
| Religiosity | 0.01 | 0.05 | 0.01 | 0.09 | 0.93 | -0.10 | 0.11 |
| Political Orientation | -0.03 | 0.04 | -0.06 | -0.88 | 0.38 | -0.10 | 0.04 |
| Note. F(12, 178) = 4.52, p < .001; R2 = .23 | | |  |  |  |  |  |
