# Supplementary material for: Individual differences in personality predict the use and perceived effectiveness of essential oils
Source: PLoS One. 2020 Mar 12;15(3):e0229779. doi: 10.1371/journal.pone.0229779 (PMC7067385; doi:10.1371/journal.pone.0229779)
Supplement: S24 Table — (DOCX) [file pone.0229779.s024.docx]

| Supplementary Table 24. Models predicting willingness to spend money on EO | | | | | | |  |
| --- | --- | --- | --- | --- | --- | --- | --- |
|  | *b* | SE | *β* | *t* | *p* | LB | UB |
| Intercept | 1.79 | 0.54 |  | 3.33 | <0.001 | 0.73 | 2.85 |
| Extraversion | 0.03 | 0.07 | 0.02 | 0.39 | 0.70 | -0.11 | 0.17 |
| Agreeableness | -0.15 | 0.08 | -0.08 | -1.88 | 0.06 | -0.31 | 0.01 |
| Conscientiousness | -0.07 | 0.08 | -0.04 | -0.87 | 0.39 | -0.22 | 0.08 |
| Neuroticism | -0.04 | 0.06 | -0.03 | -0.69 | 0.49 | -0.17 | 0.08 |
| Openness to Experience | -0.16 | 0.08 | -0.09 | -1.98 | 0.05 | -0.32 | -0.002 |
| Bullshit Receptivity | 0.39 | 0.05 | 0.26 | 7.46 | <0.001 | 0.28 | 0.49 |
| Need for Cognition | -0.03 | 0.07 | -0.02 | -0.46 | 0.65 | -0.17 | 0.10 |
| Age | 0.01 | 0.003 | 0.06 | 1.84 | 0.07 | 0.00 | 0.01 |
| Gender | 0.22 | 0.05 | 0.17 | 4.90 | <0.001 | 0.13 | 0.31 |
| Income | 0.04 | 0.02 | 0.07 | 2.04 | 0.04 | <0.001 | 0.07 |
| Religiosity | 0.12 | 0.02 | 0.20 | 5.63 | <0.001 | 0.08 | 0.16 |
| Political Orientation | -0.04 | 0.02 | -0.06 | -1.74 | 0.08 | -0.09 | 0.01 |
| Note. F(12, 753) = 24.38, p < .001; R2 = .28 | | |  |  |  |  |  |
