# Supplementary material for: Individual differences in personality predict the use and perceived effectiveness of essential oils
Source: PLoS One. 2020 Mar 12;15(3):e0229779. doi: 10.1371/journal.pone.0229779 (PMC7067385; doi:10.1371/journal.pone.0229779)
Supplement: S25 Table — (DOCX) [file pone.0229779.s025.docx]

| Supplementary Table 25. Models predicting overall effectiveness of EO | | | | | |  |  |
| --- | --- | --- | --- | --- | --- | --- | --- |
|  | *b* | SE | *β* | *t* | *p* | LB | UB |
| Intercept | 1.50 | 0.50 |  | 3.01 | 0.003 | 0.52 | 2.47 |
| Extraversion | 0.08 | 0.07 | 0.05 | 1.18 | 0.24 | -0.05 | 0.21 |
| Agreeableness | 0.07 | 0.07 | 0.04 | 0.97 | 0.33 | -0.07 | 0.22 |
| Conscientiousness | -0.01 | 0.07 | -0.01 | -0.17 | 0.86 | -0.15 | 0.13 |
| Neuroticism | 0.04 | 0.06 | 0.03 | 0.74 | 0.46 | -0.07 | 0.16 |
| Openness to Experience | -0.04 | 0.07 | -0.03 | -0.60 | 0.55 | -0.19 | 0.10 |
| Bullshit Receptivity | 0.35 | 0.05 | 0.28 | 7.40 | <0.001 | 0.26 | 0.45 |
| Need for Cognition | -0.04 | 0.06 | -0.03 | -0.69 | 0.49 | -0.17 | 0.08 |
| Age | <0.001 | 0.003 | -0.01 | -0.32 | 0.75 | -0.01 | 0.01 |
| Gender | -0.01 | 0.04 | -0.01 | -0.27 | 0.78 | -0.09 | 0.07 |
| Income | -0.001 | 0.02 | -0.002 | -0.07 | 0.95 | -0.03 | 0.03 |
| Religiosity | 0.08 | 0.02 | 0.16 | 4.16 | <0.001 | 0.04 | 0.12 |
| Political Orientation | -0.01 | 0.02 | -0.01 | -0.35 | 0.72 | -0.05 | 0.03 |
| Note. F(12, 7511) = 11.39, p < .001; R2 = .15 | | |  |  |  |  |  |
