# Supplementary material for: Individual differences in personality predict the use and perceived effectiveness of essential oils
Source: PLoS One. 2020 Mar 12;15(3):e0229779. doi: 10.1371/journal.pone.0229779 (PMC7067385; doi:10.1371/journal.pone.0229779)
Supplement: Attachment — Submitted filename: Response to Reviewers.docx [file pone.0229779.s026.docx]

**Response to Reviewers**

We would like to thank the reviewers for their thoughtful comments on the manuscript. We very much appreciate the feedback and believe that the manuscript has improved significantly as a result of the suggestions in this round of review. We are happy to make any additional changes that the Editor feels is necessary.

The line numbers below refer to the clean (i.e., not tracked changes) version of the manuscript.

**Associate Editor**

1.) The Editor noted that we should adhere to PLOS ONE’s style and naming conventions. We have now edited the manuscript so that it is consistent with the templates linked to us by the Editor.

2.) The Editor also recommended that we change “bullshit receptivity” to “receptivity to pseudo-profound fabricated statements” in the Abstract. We have now done so.

**Reviewer #1**

Reviewer #1 characterized our paper has containing a “very good study” and that the “explanation given was extraordinary.” They noted that research and publication efforts were satisfied, the introduction was great, and that the results were greatly explained.

We thank the reviewer for their kind assessment of our paper. They noted that the Discussion could be improved and we have now done so according to some recommendations made by Reviewer #2.

**Reviewer #2**

1.) Reviewer #2 recommended that we integrate the literature on the health benefits of religiosity and spirituality. Indeed, there is an extensive literature on the health benefits of religious and/or spiritual individuals (and the mechanisms linking the two). We have now added these considerations to the Introduction (on pages 4, lns 58-74) and Discussion (on pages 14-15, lns 268-281). We also make a connection to how the use of essential oils reflects an engagement with religious traditions and preventative health behaviors more generally.

2.) Reviewer #2 also recommended that we discuss some limitations related to measurement, particularly of the BSR measure and our narrow measure of religiosity (instead of a broader measure of spirituality). We have now done so in the Discussion on pages 17-18 (lns 321-346). They also recommended that we cite literature from alternative paradigms, especially those published in a particular journal (e.g., the European Journal of Integrative Medicine). Although our methodological approach substantially differs from that used in studies examining the effectiveness of EOs, we have now added some of this literature to pg 3 (lns 45-49)

3.) Finally, the reviewer noted two minor corrections (inserting the word “as” into two sentences). We have now made these changes.
